# Adhesion of Asphalt to Natural Aggregates and Sanitary Ceramic Waste

**DOI:** 10.3390/ma18061201

**Published:** 2025-03-07

**Authors:** Wojciech Andrzejuk, Agnieszka Woszuk, Danuta Barnat-Hunek

**Affiliations:** 1 Faculty of Technical Sciences, John Paul II University in Biała Podlaska, Sidorska 95/97, 21-500 Biała Podlaska, Poland; w.andrzejuk@dyd.akademiabialska.pl; 2 Faculty of Civil Engineering and Architecture, Lublin University of Technology, Nadbystrzycka 40, 20-618 Lublin, Poland; a.woszuk@pollub.pl

**Keywords:** adhesion, asphalt, sanitary ceramic waste aggregate, roughness, surface free energy

## Abstract

Research was conducted to improve our knowledge pertaining to the physical processes happening at the interfaces between solids (i.e., asphalt and aggregate) and to determine the appropriate choice of asphalt as well as additives to enhance the longevity of bituminous and mineral mixtures. The lowest mean contact angle CA with asphalt at 140 °C was obtained for dolomite and asphalt 50/70 + W (45.0°) and was 29.5% lower than the highest obtained for granodiorite and asphalt 45/80-55 (63.8°). The lowest SFE value was obtained for dolomite aggregate, it was 14.3% lower than the highest value and amounted to 47.68 mJ/m^2^. In the case of waste ceramic aggregate, the lowest mean asphalt detachment stress (0.77 MPa) was obtained using 45/80-55 modified asphalt without adhesives, at 120 °C, and it was 69.2% lower than the highest value (2.50 MPa) obtained when using samples with 45/80-55 + W modified asphalt at 160 °C. Statistically, the temperatures of both the asphalt and aggregate had the most considerable influence on the asphalt–to-aggregate adhesion, as measured by the contact angle of the aggregate with the asphalt, as well as the pull-off. The employed aggregate, as defined by its roughness characteristics, was also of importance, but to a lesser degree. The type of asphalt had the smallest impact on adhesion, but it is crucial to remember that the viscosity of asphalt is strongly influenced by temperature.

## 1. Introduction

Environmental conditions involving moisture or water are seen as the most harmful to the quality, applicability and performance of mineral and asphalt mixtures (MAMs). The existence of water or moisture in the pavement structure and its negative impacts on asphalt mixture properties can lead to diverse pavement issues, e.g., asphalt stripping, peeling, cracking and rutting.

Damage caused by moisture or water corresponds to a decrease in durability and strength of MAMs resulting from the occurrence of water or moisture in their structure [1]. The processes that lead to pavement damage are complex and occur in various forms, e.g., reduced adhesion between the aggregate and binder, the weakening of binder cohesion, the weakening of cohesion within the aggregate and the freezing of moisture retained in the pavement structure [2]. The most frequent types of moisture damage include the weakening of the aggregate and asphalt bond, as well as the weakening of cohesion within the asphalt itself [2,3,4].

In road construction, there is a phenomenon known as asphalt to aggregate adhesion. This is connected with the capacity of the asphalt binder to wet and stick to aggregate grains. The measurement of adhesion corresponds to the force that is necessary to separate the binder from the surface of the gravel for a given contact area between these materials. The main reason for road surface damage is the insufficient adhesion of asphalt to gravel grains [5,6,7]. In Modified Asphalt Mixtures (MAMs), adhesion failure occurs when the bitumen separates from the aggregate surface, showing weak adhesive bond strength. The asphalt-to-aggregate adhesion is mostly determined by the properties characterizing the materials involved, which include the chemical formula of the aggregate and asphalt, the mineralogy and aggregate texture and the amount and type of additives and fillers used. Cohesive failure occurs when there are bitumen defects resulting from its lower cohesive bond strength relative to its adhesive bond strength [8].

Numerous concepts elucidating gravel and asphalt binder adhesion are found in the literature. Among them, one can distinguish the following theories: mechanical, chemical, weak boundary layer, thermodynamic and others [9,10,11].

The mechanical adhesion theory posits that the aggregate–to-binder bond is impacted by various physical characteristics of the aggregates, including size, surface texture, shape, porosity, and absorptivity. The binder penetrates the irregularities and surface voids of the aggregate particles and solidifies, thereby establishing a mechanical bond. This theory indicates that a more robust adhesive bond is formed between asphalt and aggregates that possess a rough and porous surface.

The chemical adhesion theory was formulated to elucidate the variations in levels of adhesion among various asphalt and gravel types, particularly in the context of water and moisture presence. Aggregates can be categorized into hydrophilic, attracting water, and hydrophobic, repelling it. Chemical surface reaction, porosity and pore size are the main properties of aggregates determining the group they fall into, i.e., hydrophilic or hydrophobic [3,12]. In general, aggregates with higher acidity are less likely to form strong adhesive bonds with asphalt, which later affects the vulnerability of the MAM [13].

The thermodynamic adsorption theory serves as the predominant framework for elucidating interfacial adhesion. This theory posits that the binder adheres to the substrate as a result of the physical interfacial forces of the two phases, following their contact. The strength of characterizing these forces is typically associated with essential thermodynamic parameters, including the surface energy associated with the materials present in each phase [14]. To ensure a robust asphalt-to-aggregate bond, the asphalt must be thoroughly covered, or the surfaces of the aggregate particles wetted.

The theory of molecular orientation posits that upon asphalt and gravel surface interaction, the molecules of asphalt align themselves in a manner that satisfies the energy requirements of the aggregate surface.

The weak boundary layer theory posits that adhesive bonds may experience failure within the binder or aggregate when an interfacial region characterized by low cohesion strength is present [15,16]. Additionally, the electrostatic theory, as discussed by Dallas et al. [17], suggests that solid surfaces may be classified as either electronegative or electropositive. This classification arises from the arrangement of atoms that exhibit an electronegative nature, leading to the formation of molecular dipoles. Consequently, the adhesion force may be understood as the force necessary to separate charged surfaces by counteracting Coulombic forces [15,16].

These theories of adhesion and their corresponding mechanisms are interrelated, with a consensus among many researchers that multiple mechanisms operate concurrently in MAMs.

Given the complexity of the various adhesion theories, for the sake of simplicity, the factors influencing asphalt-to-aggregate adhesion include mechanical adhesion and physicochemical factors. Mechanical factors include the following: the degree of moisture in the aggregate (a layer of water on aggregate surface hinders the asphalt from adequately enveloping the grains); the dustiness of the aggregate—when dust is present on the aggregate grains, it obstructs the penetration of asphalt into the aggregate; and the micro-texture of the aggregate grains—a better micro-texture results in a larger surface area for the asphalt to adhere to the aggregate. The physicochemical factors include the degree of acidity of the aggregate; the mineralogical composition of aggregates significantly influences the adhesion between the asphalt and aggregate. The hierarchy of adhesion strength between aggregates and asphalt is established in the following manner: alkaline aggregates exhibit the highest strength, followed by neutral aggregate and finally acidic aggregate. CaCO_3_, occurring in limestone, provides Ca^2+^ ions, which are essential for enhancing the adhesion force between limestone and asphalt [18].

Asphalts have a higher affinity for alkaline rocks compared to acidic ones. In the aggregate, a reduced silica content enhances the adhesion of the asphalt [19]. This is attributed to the chemical and physical characteristics of asphalt, which typically contains a higher proportion of acidic compounds, thereby improving its adhesion to alkaline aggregates.

In addition to the factors mentioned above, adequate adhesion also requires that the asphalt has a sufficiently low viscosity, which forces it to be heated to high temperatures [5,20]. Dynamic viscosity serves as a crucial rheological parameter for asphalt, profoundly influencing its mechanical and physical properties, in addition to the performance of asphalt pavement structure layers. With a sufficiently low viscosity at high temperatures, asphalt surrounds the aggregate grains well during the manufacturing stage of mineral–asphalt mixtures [21]. Temperature greatly influences the viscosity of asphalt.

Numerous scholars have addressed the topic of asphalt and aggregate adhesion in their scientific work, using both experimental methods and computer simulations.

F. Pan et al. [22] conducted an analysis of the adhesion properties of granodiorite, dolomite and limestone to bitumen, assessing how various mineral components influence adhesion. Their findings indicated that dolomite and limestone exhibited superior interfacial adhesion to asphalt compared to granodiorite. Furthermore, titanium and calcium oxides demonstrated the greatest adsorption potential on bitumen. Additionally, elevated calcium oxide levels enhanced the adhesion of asphalt to limestone and dolomite, as opposed to granodiorite, a conclusion that was further substantiated by supplementary analysis of the particle concentration profiles.

Also, Y. Hou et al. [6] conducted research related to adhesion at the interface of gravel and asphalt. The study conducted an investigation into the adhesion properties of recycled concrete aggregate (RCA) with asphalt. It involved testing the CA of droplets, specifically ethanol and water, in relation to natural aggregates and RCA, as well as solid bitumen (including SBS-modified asphalt and matrix asphalt) through a droplet method utilizing optical microscopy. Subsequently, the contact angles between hot asphalt and both RCA and natural aggregates were calculated, in addition to their surface free energy (SFE). The findings revealed that a high proportion of RCA in the mineral mix could result in the increased porosity of hot mix asphalt (HMA-RCA). Furthermore, the contact angle and adhesion energy between the RCA and asphalt markedly influenced the fatigue characteristics and water stability of the HMA-RCA.

A. Su et al. [23] evaluated the impact of the type of aggregate on the interface of asphalt and gravel by examining adhesion at three distinct scales. Their macroscale analysis involved a pull-off test performed on asphalt in conjunction with three different types of aggregate, allowing for a direct assessment of their adhesion. At the microscale, the surface energy of asphalt was studied in relation to limestone, basalt and andesite; in addition, the work of adhesion was assessed. At the nanoscale, dynamics simulations were deployed to study the behavior at the interface between six mineral compositions (Na_2_O, Fe_2_O_3_, Al_2_O_3_, MgO, CaO and SiO_2_) present in the asphalt and aggregates. Additionally, the study explored the relationship between adhesion work at the nano- and microscale to elucidate the aggregate–asphalt bonding mechanisms. The findings at the macroscale indicated that the andesite exhibited higher surface roughness compared to limestone. The findings obtained on the microscale revealed that superior water stability and adhesion were observed between limestone and asphalt. This discovery also highlighted that alkali aggregates demonstrated the strongest adhesion at the interface with asphalt. At the nanoscale, the adhesion between asphalt and alkali oxides was found to surpass that of acid oxides. Furthermore, basalt and limestone displayed enhanced adhesion to asphalt as a result of their greater content of alkali oxides, whereas andesite showed inferior adhesion, owing to its lower alkali oxide content as well as elevated SiO_2_ levels.

The research conducted by L. Li et al. [24] examined the impact that the lithology of aggregates, chemical ingredients of asphalt and the morphology of aggregates on adhesion. They employed a non-contact, three-dimensional (3D) white light scanning method to gather point cloud data regarding the aggregate particles. Their analysis focused on how aggregate morphology and the chemical ingredients of asphalt affect adhesion levels, as well as the compatibility of various lithological aggregates with asphalt. The findings indicated that the asphalt and aggregate adhesion is characterized by both physical and chemical adsorption; the chemical bond is notably robust, whereas the mechanical locking forces and physical orientation are comparatively weaker. For asphalts containing a high proportion of colloidal aggregates, it was recommended to utilize alkaline lime aggregates. Conversely, for asphalts with elevated colloid content, basalt aggregates with low alkalinity should be preferred.

The presented research aimed to describe the factors affecting the adhesion of asphalts to natural and artificial aggregates by determining the CA, SFE, work of adhesion (WA) and adhesion. Relationships and correlations were formulated to enable the interpretation of the results of the studies on the properties of adhesion.

The objective of this study was to determine the physical processes taking place at the interfaces of solid phases (aggregate–asphalt) and to appropriately select the additives and asphalt types to enhance the strength of MAMs that also incorporate recycled aggregates.

The findings regarding sanitary ceramic waste aggregate indicate that this recycled material has the potential to serve as a replacement for natural aggregates utilized in MAMs, which promotes sustainability in road construction and reduces the amount of construction waste that goes to landfills.

## 2. Materials and Methods

### 2.1. Asphalts

The tests and mixes utilized two varieties of asphalt: road asphalt 50/70 as well as polymer-modified asphalt PMB 45/80-55. The parameters selected for testing these binders are detailed in Table 1.

### 2.2. Adhesion Agents

The asphalts used in the study came in two variants: with and without the addition of an adhesion agent. In addition, in determining the characteristics of the asphalts and evaluating their adhesion quality, selected surfactant formulations were also used. The adhesion agents utilized included Wetfix BE and Rediset LQ-1102CE, sourced from Nouryon global manufacturing facilities (previously known as Akzo Nobel), along with SBS Polymer for Asphalt Modification, provided by LG Chemicals in Korea. The test was performed using the dyna pull-off tester Z-25 from Proceq (Switzerland, Schwerzenbach).

#### 2.2.1. Wetfix BE

Wetfix BE adhesion agent for road asphalts is utilized to enhance the adhesion of asphalt binders used for road construction and maintenance (increases the asphalt to aggregate adhesion). The product is recommended both as an additive to ordinary and modified asphalts.

The properties of Wetfix BE are as follows: acid value < 10 mgKOH/g; amine value 246–285 mg KOH/g; liquid at 25 °C; flash point > 218 °C; pour point < −20 °C; density 0.98 g/cc at 20 °C; viscosity 538 mPa·s (cP) at 20 °C [25].

#### 2.2.2. Rediset LQ-1102CE

Rediset LQ-1102CE is an agent that improves the compaction of hot and warm mixes. It allows for the lowering of the mixing and paving temperatures while preventing asphalt from washing off the aggregate surface. The properties of Rediset LQ-1102CE are as follows: amine value 540–640 mg KOH/g; water max.1%; liquid at 25 °C; flash point > 165 °C; pour point 0 °C; density 1.0 g/cc at 20 °C; viscosity 1703 mPa·s (cP) at 20 °C; dosage to hot and warm mix 0.3–1.0%. [26].

### 2.3. Aggregates

#### 2.3.1. Traditional Aggregates

The study incorporated a range of natural aggregates that are conventionally utilized in asphalt pavements. Notably, these aggregates comprised dolomite sourced from the Polish Dolomite Mine in Piskrzyn and granodiorite obtained from Tomashgorod Rokytne in Rivn, Ukraine. All the aggregates utilized conform to the standards required for aggregates in bituminous mixtures, as well as surface treatments applicable to airports, roads and other traffic-related surfaces.

A summary of the selected measured parameters for the tested aggregates is provided in Table 2.

#### 2.3.2. Recycled-Waste Aggregate

Alongside the conventional natural aggregates typically employed in MAMs, this study also incorporated recycled-waste ceramic aggregate sourced from a refuse pile at a sanitary ware manufacturing facility. The materials primarily consisted of sanitary ware items exhibiting cracks, flawed glazes, or irregular surfaces. This waste underwent a crushing process utilizing jaw crushers.

The basic technical properties of this aggregate obtained from the authors’ own tests are presented above (Table 2).

A detailed assessment of recycled ceramic aggregates in terms of their potential use in mineral–asphalt mixtures was carried out by the authors of this manuscript in another article [27].

### 2.4. Studies

**Figure 1:**
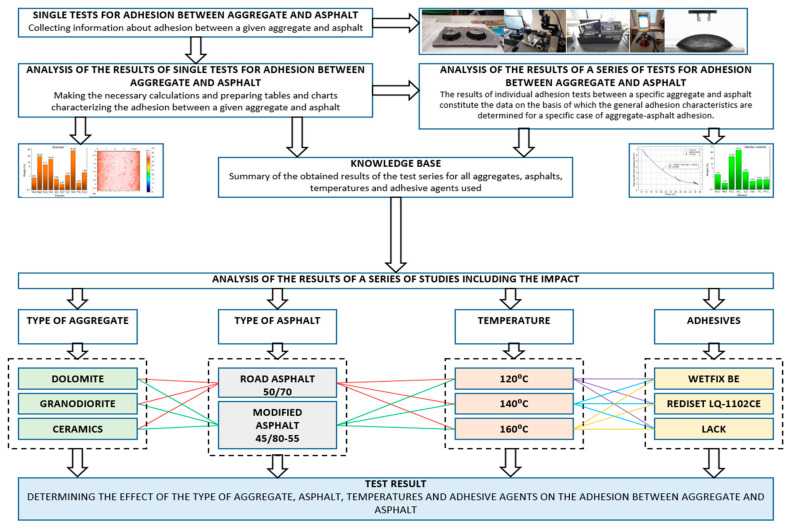
A diagram illustrating the analysis of the results obtained from the conducted tests on adhesion, correlated with the durability of the MAMs.

The evaluation of the outcomes from the conducted tests was performed in accordance with the methodology depicted in Figure 1.

The study was carried out to establish the impact of the type of aggregate, asphalt, temperature and adhesion agents on the adhesion between the former two.

### 2.5. Microstructure and Elemental Analysis of Aggregates

A scanning electron microscope from FEI in Hillsboro, OR, USA, was utilized for this study. This microscope is equipped with a chemical composition analysis system that employs energy dispersive X-ray spectroscopy (EDS), to analyze the structure and morphology of materials.

### 2.6. Aggregate Surface Geometry—Roughness Parameters

A modular Hommel-Etamic T8000 RC120-400 device was employed to assess surface roughness and three-dimensional topography. The analysis of roughness characteristics was performed through the standardized user interface, which facilitates the computation of all recognized roughness parameters as well as the evaluation of geometric attributes, e.g., radii, angles and distances [28].

The scope of the study included measuring the roughness of the granodiorite aggregate, dolomite aggregate and waste ceramic aggregate. The tested samples had their surfaces tested in a dry and wet state.

The study was performed based on the following parameters: stylus type: 2 μm (radius); stylus force: 5 mg; duration: 40 s; resolution: 0.25 μm/pt; map resolution: 5 μm/trace.

Since crushed aggregate was used in the tests, fragments of separated grains of individual aggregates were randomly selected to determine their roughnesses, measuring the surface roughness of 4.5 × 4.5 mm^2^ samples.

### 2.7. Dynamic Viscosity

Dynamic viscosity measurement was performed in compliance with PN-EN 13302 [29]. This assessment utilized the Brookfield AMETEK Thermosel system, which includes a viscometer (DV3T) along with the necessary accessories to ensure precise viscosity measurements of fluids at high temperatures.

PN-EN 13302 was adopted to measure dynamic viscosity [29]. A Brookfield AMETEK viscometer (DV3T) for accurate measurement of fluid viscosity at elevated temperatures were used for testing.

Dynamic viscosity was measured at t_1_ = 120 °C, t_2_ = 140 °C and t_3_ = 160 °C. At 120 °C, the shear rate was 20 RPM (revolutions per minute), at 140 °C it was 100 RPM and at 160 °C it was 200 RPM. To ensure the validity of the results, six readings were taken from each sample at the tested temperature.

### 2.8. Determination of Affinity Between Aggregate and Asphalt—Rotated Bottle Method

To assess the adhesion between gravel and the asphalt binder, the affinity between the asphalt and aggregate was evaluated by means of the rotated bottle method, as outlined in PN-EN 12697-11:2020-07 [30]. This procedure involves visually examining the asphalt coating on uncompacted aggregate that has been mixed mechanically with water, allowing for the determination of adhesion based on the appearance of the asphalt layer.

### 2.9. The Determination of the Contact Angle of Aggregates with Glycerin and Water

The contact angles (CAs) of liquid drops were assessed using a testing apparatus that included an optical goniometer (OCA 25 DataPhysics Instruments, Bengaluru, India) and a camera for capturing images of individual drops placed on the surface of each sample [31]. The contact angles were analyzed with glycerin and distilled water. The measurements of contact angles, θw, associated with the surface coatings, were carried out using a liquid with known total SFE values (γw). A total of twelve drops were deposited onto each sample, with measurements taken at a controlled temperature of 23.0 ± 1.0 °C at the moment of drop application.

The aggregate samples, shaped as 3.0 × 4.0 cm tiles and prepared using a stone-cutting machine, were utilized for the experiments. Each tile was allowed to rest in a controlled environment for 24 h to ensure the stabilization of its surface free energy. Subsequently, the tiles were cleaned with deionized water, dried at 105 °C and then left to cool to ambient temperature.

A drop of the test liquid was dosed onto the surface of the tested aggregates using an automatic micropipette dosing system. It was important that the drop had the right volume; too small could lead to measurement errors, while too large could cause it to flatten under the influence of gravity. A drop with a volume of 2 mm^3^ was used in the present study.

When the drop was placed on the surface of the tested aggregate, the CA was measured using a goniometer. This device recorded the image of the drop and analyzed its profile, determining the wetting angle as the tangential angle between the surface of the solid and the tangent to the contour of the drop at the point of contact with the substrate.

The wetting angle was determined automatically using image analysis algorithms.

### 2.10. The Determination of the Contact Angle of Aggregates with Asphalt

The apparatus and samples prepared in the same way as in 2.9 were used in the study.

The experiment to determine the contact angle of the aggregates with asphalt was conducted at three different temperatures (120 °C, 140 °C, 160 °C), which directly affected the viscosity conditions of the asphalt.

The aggregate samples were previously heated to the required temperature, which was then maintained during the test by the goniometer’s environmental chamber. Similarly, the asphalt drops were dosed at the appropriate temperature using an automatic dosing system with the possibility of heating.

Similarly to point 2.9, the wetting angle was determined automatically using image analysis algorithms.

### 2.11. The Surface Free Energy (SFE) in the “Aggregate–Water” and “Aggregate–Water/Glycerin” Systems

The procedure for determining the surface free energy was based on measurements of the wetting angles of various liquids on the tested aggregate surfaces and on the use of an appropriate mathematical model to calculate this quantity.

Two models were used to calculate the SFE: the Neumann model [32] and the Owens–Wendt model [33]. They represent some of the most popular methods for calculating SFE, and the results obtained with them were of similar scatter. The Neumann model uses the following equation:

(1) $$cos\theta_{w}=\left[e^{-0.000125(\gamma_{s}-\gamma_{w}{)}^{2}}2\sqrt{\frac{\gamma_{s}}{\gamma_{w}}}-1\right]$$

where

γ_s_—the total SFE value;

γ_w_—the SFE of distilled water;

θ_w_—the water contact angle;

while in the Owens–Wendt model, the following equations are used:

(2) $$\gamma_{S}=\gamma_{S}^{d}+\gamma_{S}^{p}$$

(3) $${(\gamma }_{S}^{d}{)}^{\frac{1}{2}}=\frac{\gamma_{g}\left(cos\theta_{g}+1\right)-\gamma_{w}(cos\theta_{w}+1)\sqrt{\frac{\gamma_{g}^{p}}{\gamma_{w}^{p}}}}{2(\sqrt{\gamma_{g}^{d}}-\sqrt{\frac{\gamma_{g}^{p}\gamma_{w}^{d}}{\gamma_{w}^{p}}})}$$

(4) $$(\gamma_{S}^{p}{)}^{\frac{1}{2}}=\frac{\gamma_{w}\left(cos\theta_{w}+1\right)-2\sqrt{\gamma_{S}^{d}\gamma_{w}^{d}}}{2\sqrt{\gamma_{w}^{p}}}$$

where

γ_g_—the SFE of glycerin;

γ_g_^d^—the dispersive component of the SFE of glycerin;

γ_g_^p^—the polar component of the SFE of glycerin;

γ_w_—the SFE of water;

γ_w_^d^—the dispersive component of the SFE of water;

γ_w_^p^—the polar component of the SFE of water;

θ_g_—the wetting angle of glycerin;

θ_w_—the wetting angle of water.

### 2.12. The Work of Adhesion (WA) in the “Aggregate–Water” and “Aggregate–Water/Glycerin” System

The equation below was employed for calculating WA:

(5) $$W_{A}=\gamma_{s}+\gamma_{l}-\gamma_{sl}$$

where

γ_s_—the SFE of the solid;

γ_l_—the SFE of the liquid;

γ_sl_—the SFE of the solid–liquid interface.

### 2.13. Adhesion of Asphalts to Aggregate as a Function of Temperature (Pull-Off)

**Figure 2:**
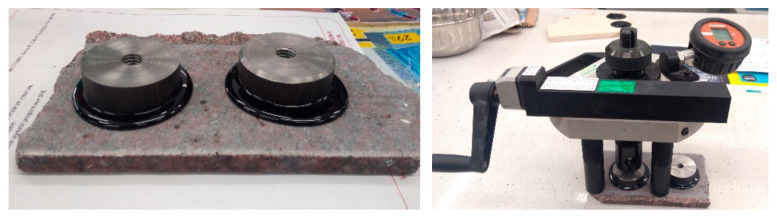
Determination of adhesion of asphalts to aggregate (granodiorite) using pull-off method.

The asphalt binder to aggregate adhesion was assessed by means of the pull-off test, which is categorized as a semi-destructive testing method. In this study, the dyna pull-off tester Z-25 instrument (refer to Figure 2) was employed for the preparation and evaluation of the samples.

First, the aggregate samples were subjected to drilling using a 50 mm drill to a 10 mm depth. A film of binder heated to the appropriate temperature was applied (at the drill spot) to the dried and heated aggregate, and then the steel ring used in the pull-off test was glued to it. The samples, thus prepared, were maintained for 24 h at room temperature. Following this interval, the peel test was conducted. The asphalt binder successfully adhered to both the surface of the aggregate and the underside of the steel rings, which measured 50 ± 0.5 mm in diameter and 20 mm in thickness. The specimen was exposed to tensile forces until it reached the point of failure. The maximum stress endured by the specimen prior to failure was recorded as the adhesion force in the pull-off test. The asphalt binders were subjected to the pull-off test at three distinct temperatures (120 °C, 140 °C, 160 °C).

### 2.14. Statistical Analysis of Research Results

In order to determine probability distributions, all study variables were subjected to statistical analysis using basic descriptive statistics and box-and-whisker plots. In addition, a comparative analysis was applied to evaluate the importance of the impacts of each independent variable and their interaction effects.

Given the fact that the analyzed dependent variable was expressed by two values, multivariate analysis of variance (MANOVA) was first applied, with the prior verification of the assumptions. Since both the assumption of the multivariate normality of the features in the subgroups and the homogeneity of the covariance matrix were not met, the permutational equivalent of the parametric MANOVA test [34] was applied. It allowed the testing of marginal effects and interaction effects. The Shapiro–Wilk test was used to test multivariate normality, while the homogeneity of the covariance matrix was verified by the Box test [35]. To identify differences between groups in terms of adhesion, permutational equivalents of multivariate multiple comparison tests [36] with Benjamini and Hochberg correction [37] were also used. To verify the significance of the differences in individual adhesion components, Welch’s univariate parametric tests for variables with heterogeneous variance were used. In the case of several comparisons, the ANOVA test assumptions were met; then, the classic one-way analysis of variance test was used. Post hoc verification was performed with the Games–Howell test (in the case of failure to meet the assumption of homogeneity of variance).

Completely independently, a cluster analysis was conducted to determine groups of observations that were homogeneous by adhesion parameters. It utilized an unsupervised machine learning method to characterize the obtained groups due to the composition of the mixture. The cluster analysis was performed in two stages [38]. The initial phase, employing hierarchical cluster analysis, aimed to identify the most suitable number of clusters. Clustering was conducted utilizing Ward’s method in conjunction with the Euclidean metric [39]. In the second stage, the k-means method was used, which divided the observations into homogeneous groups [40,41]. The results of object grouping were presented on a dendrogram and a projection diagram on the space defined by the principal components using the PCA method [42,43].

## 3. Results

### 3.1. Macrostructure and Elemental Analysis of Aggregates

**Figure 3:**
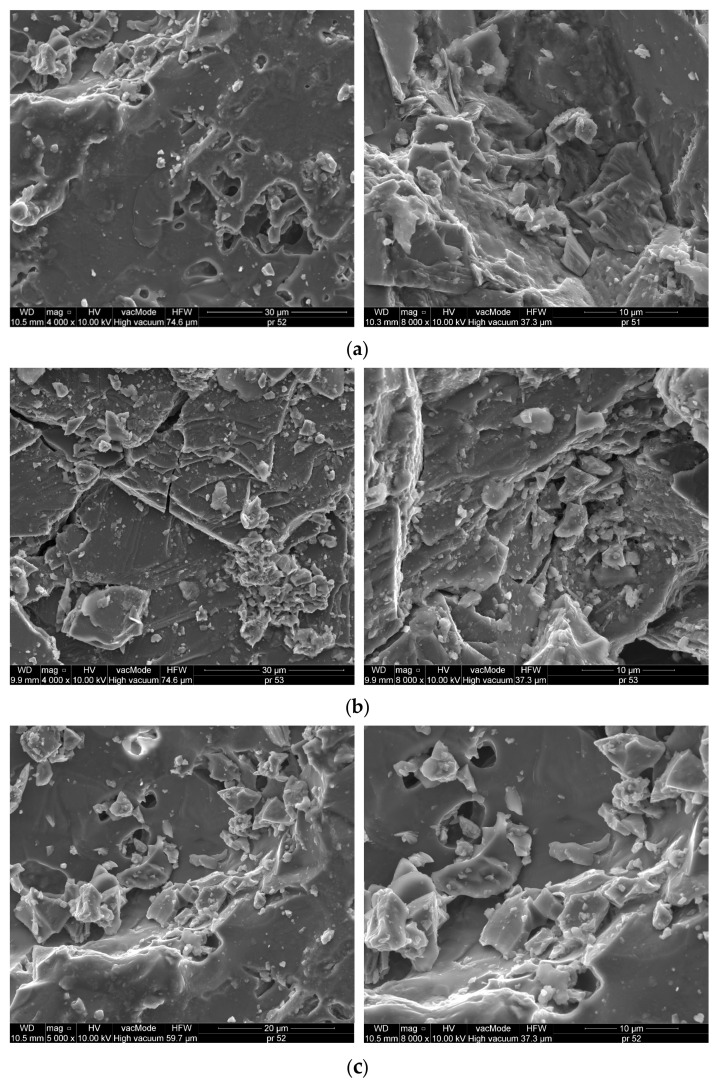
SEM microstructure of (**a**) dolomite (5000× and 8000×), (**b**) granodiorite (4000× and 8000×), (**c**) waste sanitary ceramic aggregate (5000× and 8000×).

Figure 3 illustrates the SEM images that depict the microstructure of the aggregates (raw material) under examination: dolomite, granodiorite and waste ceramic aggregate.

**Figure 4:**
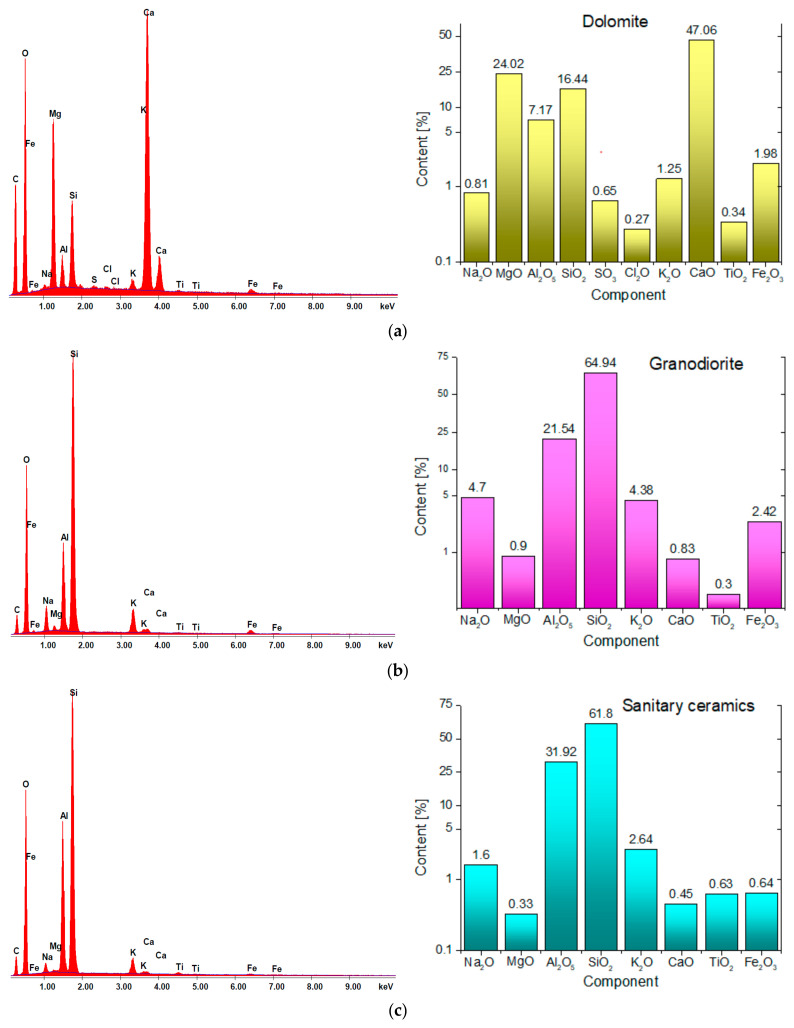
Results of EDS micro-area elemental analysis: (**a**) dolomite, (**b**) granodiorite, (**c**) waste aggregate from sanitary ceramic.

Energy dispersive X-ray spectrometry (EDS) was adopted in order to determine the chemical composition of the analyzed materials (Figure 4).

Scanning microscopy images reveal that the dolomite exhibits a highly compact structure, devoid of any visible cracks. EDS analysis indicates that the predominant components of the dolomite are calcium oxide (47.06%) as well as magnesium oxide (24.02%). Silicon dioxide (SiO_2_) measured at 16.44%, which categorizes the dolomite as an alkaline aggregate [44,45]. In contrast, the structures of the granodiorite and sanitary ceramic display slight variations, with observable voids, and granodiorite specifically shows the presence of cracks (Figure 3b). Pores with diameter greater than 5 µm were identified in both the ceramic and dolomite aggregates (Figure 3a,c), whereas the granodiorite aggregate contained significantly smaller pores, approximately 1 μm in diameter. The abundance of larger pores contributes to the increased absorbency of ceramics and dolomite when compared to granodiorite [46].

The dominant components in the case of granodiorite and sanitary ceramic are silica, with its content exceeding 61%, and Al_2_O_5_—21.5% for granodiorite and 31.9% for sanitary ceramic, respectively (Figure 4). Therefore, according to the literature, these aggregates can be considered acidic [44,45]. In the analysis of the sanitary ceramic it was noted that its Fe_2_O_3_ content was significantly lower, by a factor of 3.5 to 4, in comparison to the other aggregates examined.

### 3.2. The Geometry of the Surface of the Tested Aggregates

Since the issue of adhesion between asphalt and aggregate involves the interaction of two surfaces, we decided to perform a three-dimensional analysis of the aggregate surfaces.

**Figure 5:**
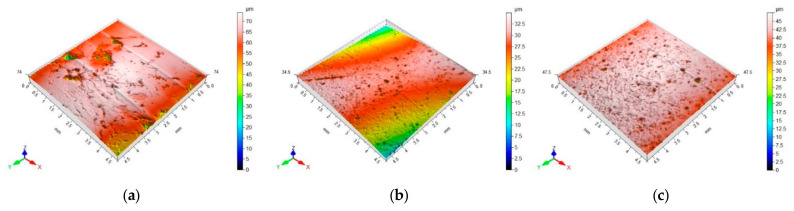
Representative 3D profilograms of the surfaces of the studied aggregates: (**a**) dolomite, (**b**) granodiorite, (**c**) sanitary ceramic.

A total of six randomly chosen sections of aggregate samples were analyzed, focusing on the roughness of the 4.5 × 4.5 mm^2^ surface area. The surface structure images captured using the 3D profilographometer illustrate the roughness that characterizes the surfaces of the aggregates under examination (Figure 5).

In order to comprehensively, graphically and numerically represent the geometric structure characteristics of the surface of aggregates in 3D, the parameter of the mean value of the arithmetic mean height, Sa, was used.

The average value of the arithmetic mean *Sa* height is the largest for dolomite (4.65 µm), while it was the smallest for ceramic aggregate (1.75 µm).

**Figure 6:**
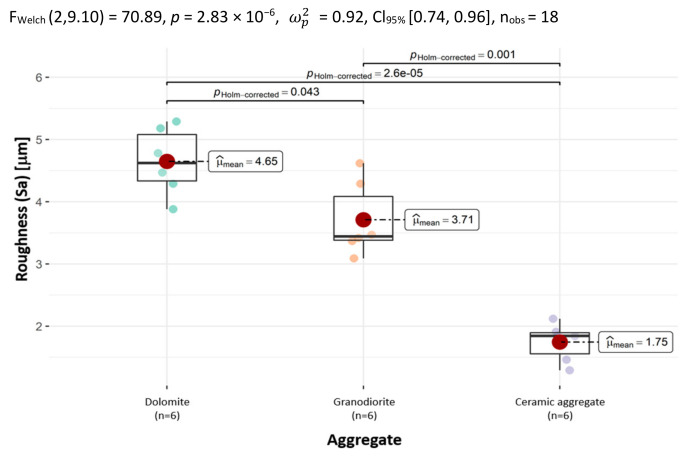
The distribution of the arithmetic mean height (Sa) according to the type of aggregate, with the determination of statistically significant differences.

To facilitate subsequent comparative analysis, a chart confirming the differences between the aggregates in terms of *Sa* is presented below in Figure 6. It shows that the differences are statistically significant.

### 3.3. Determination of Dynamic Viscosity of Binders

**Figure 7:**
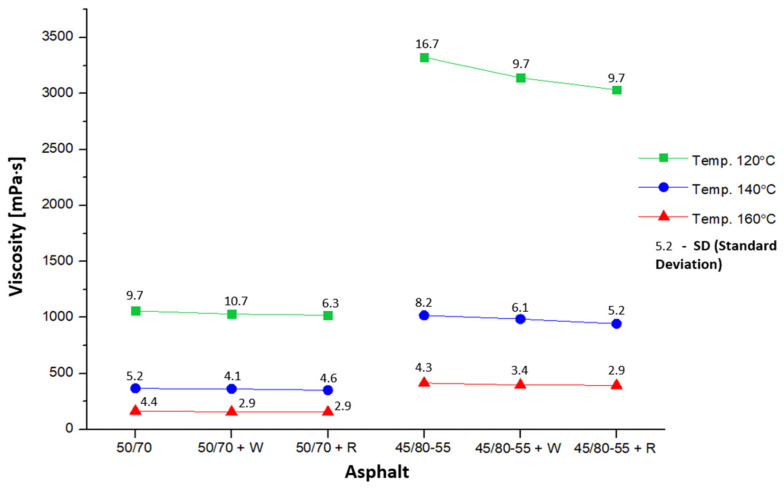
Distribution of mean dynamic viscosity values depending on temperature and type of asphalt.

In the study, combinations of asphalts were used, as shown in Figure 7. The amount of adhesion agent Wetfix BE or Rediset LQ-1102CE incorporated into the asphalt was 0.3% by weight of the binder.

Six samples of each of the tested asphalts were used for testing. The dynamic viscosity of the binders was measured at three distinct temperatures. The results pertaining to the dynamic viscosity of the evaluated binders are presented in Figure 7.

In addition, Figure 7 also illustrates the boundary effects of asphalt and temperature as well as their interaction on the dynamic viscosity outcomes of the asphalts employed in the study. It can be observed how temperature affects viscosity, and that the two different types of asphalts, 50/70 and 45/80-55, have different viscosity levels over a range of set temperatures. The interaction effect is also significant, as evident by the changing temperature that modifies the effect of asphalt type on viscosity.

On the basis of the results obtained, it is possible to observe compliance with the standard tendency for asphalt binders to decrease viscosity values as the test temperature increases [47]. This trend applies to the full range of temperatures used in the experiment. In addition, it can be stated that it is possible to adjust the viscosity of asphalt using additives [48]. In this specific instance, the incorporation of an additive as an adhesive agent at a concentration of 0.3% by weight of the binder leads to a minor decrease in the dynamic viscosity measurements across both categories of asphalts and at all tested temperatures. Thus, the lowest viscosity value at 120 °C was obtained by asphalt 50/70 + R, and it was 3.7% lower than the maximum value observed for asphalt 50/70. The same is true in the group of modified asphalts; binder 45/80-55 + R obtained an 8.7% lower viscosity value than 45/80-55. At 140 °C, in the group of ordinary asphalts, the lowest viscosity was shown by binder 50/70 + R, and it was 4.2% lower than the value obtained by asphalt 50/70. Similarly, in the group of modified asphalts, asphalt 45/80-55 + R obtained a 7.2% lower viscosity value than 45/80-55. At 160 °C, in the first group, asphalt 50/70 + R recorded a viscosity value 5.8% lower than 50/70, while in the second group, binder 45/80-55 + R obtained a viscosity value 4.8% lower than 45/80-55.

### 3.4. Determination of Affinity Between Aggregate and Asphalt

**Figure 8:**
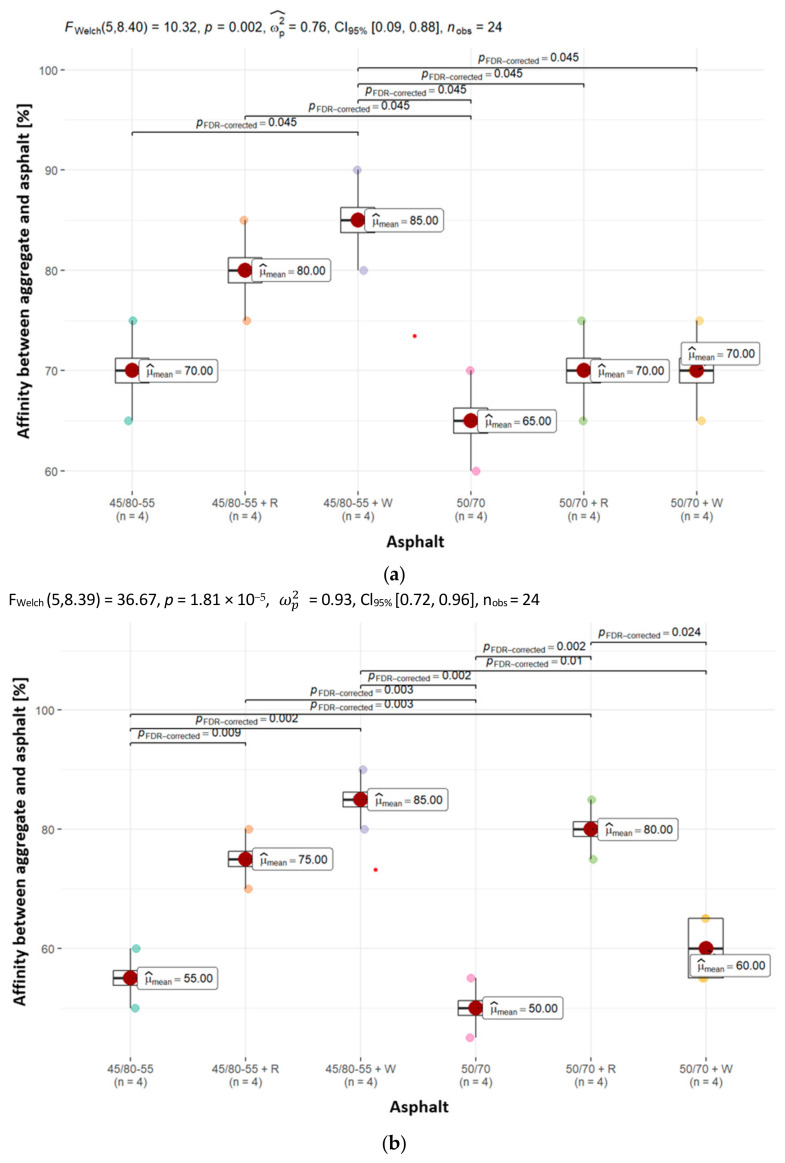

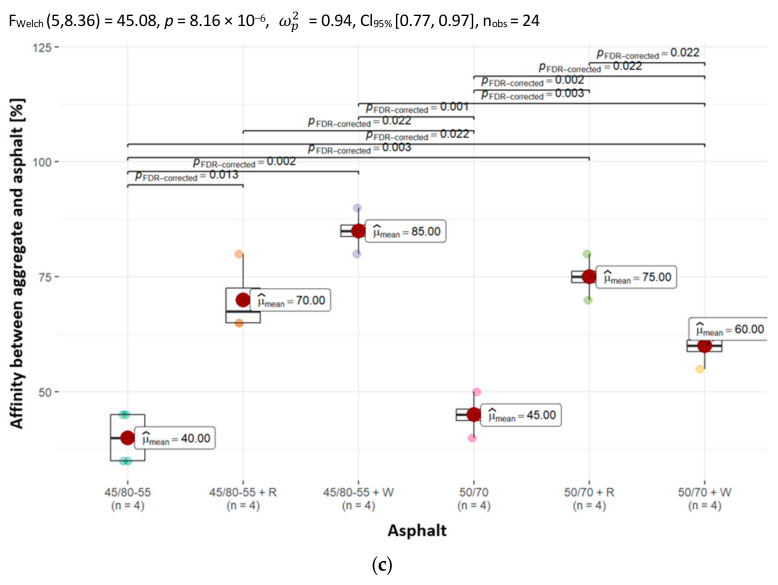
The affinity between (**a**) dolomite, (**b**) granodiorite and (**c**) ceramic aggregate and the asphalts analyzed in the paper, together with the determination of statistically significant differences.

To evaluate the asphalt coverage of uncompacted aggregate grains after subjecting them to mechanical mixing in water, an affinity (adhesion) determination was carried out between the analyzed aggregates (dolomite, granodiorite, sanitary ceramic) and asphalts (50/70, PMB 45/80-55). The experiment was carried out in line with PN-EN 12697-11, method A. The evaluation of binder washout from the aggregate was performed after 6 h, and the findings, expressed in percentages, are presented in Figure 8.

The results of testing the affinity of asphalts without adhesives to aggregates are illustrated through the chemical composition analysis of the aggregates. The mean affinity is highest for the alkali rock dolomite, amounting to 65% and 70% (for asphalt 50/70 and 45/80-55, respectively) after 6 h of testing (Figure 8). The weakest unsatisfactory affinity was obtained for aggregates with much higher silica content—granodiorite and ceramic aggregate. In the case of granodiorite, its mean affinities were 50% and 55% (for asphalt 50/70 and 45/80-55), respectively, and in the case of ceramic aggregate, its mean affinities equaled 45% and 40% (for asphalt 50/70 and 45/80-55), respectively.

The mean affinity values between the aggregates used in the study and the asphalts with 0.3% adhesion agents are different. In all types of aggregates and asphalts, a clear increase in the values of their mean affinity can be noted, though it is important to emphasize that not every adhesion agent improves adhesion to the same extent, and this is confirmed by the results obtained. The largest increase was recorded for the acidic aggregates, namely granodiorite and ceramic aggregate. In the case of the latter, an average increase in affinity of up to 45% was registered. It can also be observed that the mean affinity when using asphalts with adhesive agents is higher when using modified asphalt (45/80-55 + W, 45/80-55 + R). In addition, in many cases, the adhesion of asphalt to aggregate at a minimum of 80% after 6 h of conducting the “rotating bottle” test was achieved (e.g., dolomite and 45/80-55 + W, granodiorite and 50/70 + R, granodiorite and 45/80-55 + W, ceramic aggregate and 45/80-55 + W).

The results obtained indicate that the incorporation of adhesives into asphalts significantly enhances the affinity values between all the tested aggregates and asphalt groups analyzed in this study.

### 3.5. CA Determination of Aggregates with Water and Glycerin

**Figure 9:**
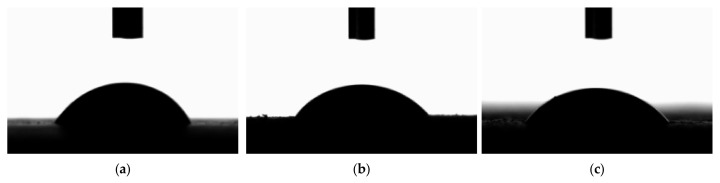
Water droplet wetting measurements of selected aggregates: (**a**) dolomite, (**b**) granodiorite, (**c**) ceramic aggregate.

In assessing the wettability of the aggregates used in the study, CA measurements were carried out. Distilled water and glycerin, two liquids with known SFE parameters— which are total SFE, its dispersive and polar components—were used for testing. A micropipette was utilized to deposit fixed volumes of liquid droplets of about 2 mm^3^ onto the sample surface. Twelve CA readings were taken on each aggregate. Figure 9 shows examples of the images used to determine CA obtained during the tests.

**Figure 10:**
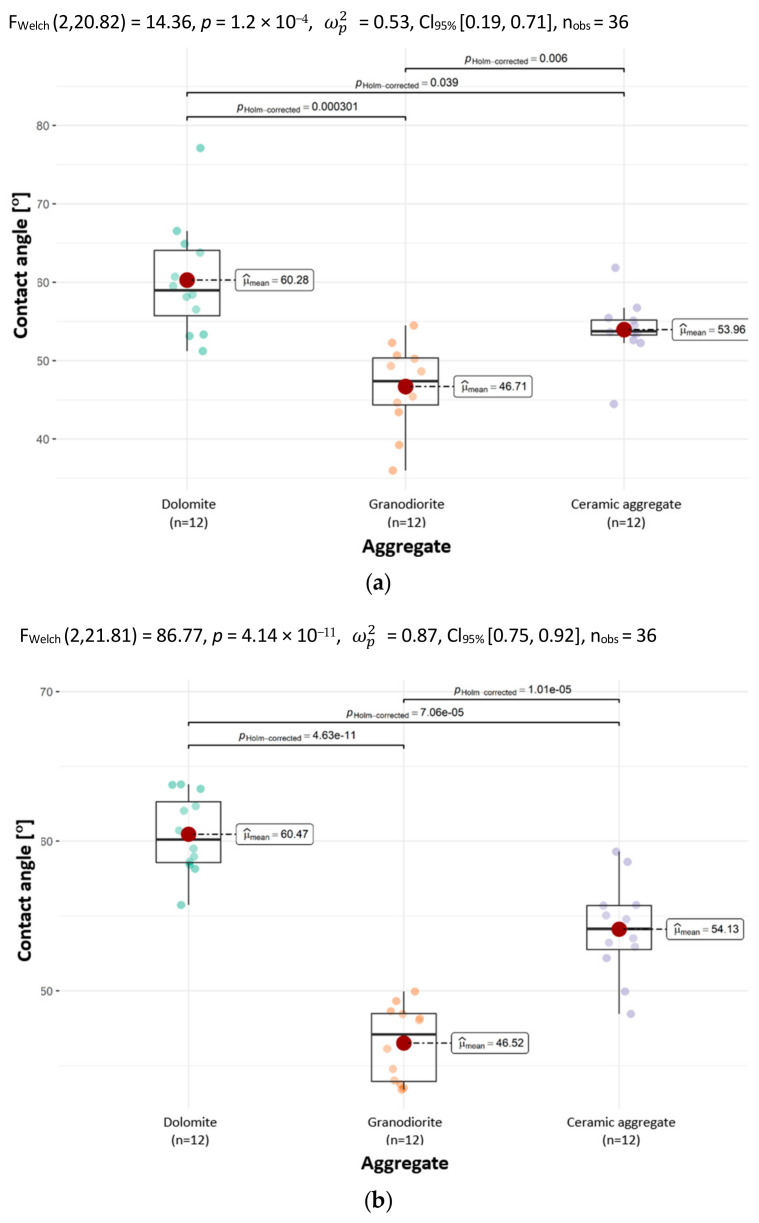
The contact angles of the tested aggregates with the determination of statistically significant differences with a drop of (**a**) distilled water and (**b**) glycerin.

The graphs in Figure 10 suggest that all the analyzed aggregates are statistically significantly different in terms of CAs with distilled water and glycerin.

The results indicate that CA values are dependent on the aggregate type. The findings pertaining to CA with water showed that the mean CA of dolomite is higher than the mean CA of granodiorite and ceramic aggregate. The highest mean CA for water was observed on dolomite and was 60.28°. The smallest mean CA for water was obtained on granodiorite; it was smaller than the largest by 22.5% and was 46.71°.

For the CAs of aggregates with a drop of glycerin, the results were analogous to the CAs with water. The mean CA of dolomite was larger than that of granodiorite and ceramic aggregate. The highest mean CA using a drop of glycerin was observed on dolomite and was 60.47°. The smallest mean CA with glycerin was obtained on granodiorite, it was smaller than the largest by 23.1% and was 46.52°.

### 3.6. Determination of Contact Angle of Aggregates by Asphalt

**Figure 11:**
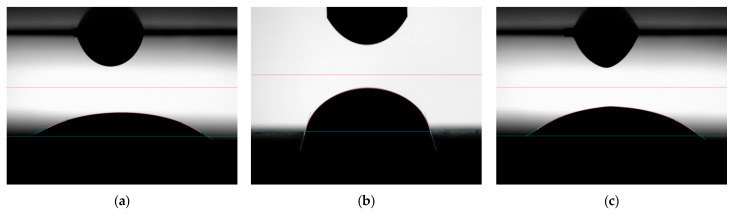
Selected photos of aggregate wetting with asphalt: (**a**) dolomite, (**b**) granodiorite, (**c**) ceramic aggregate.

The determination of the wettability of the aggregates by asphalt was intended to simulate the actual contact of the aggregate–asphalt system occurring during the mixing of MAMs. The experiments were carried out at three temperatures: 120 °C, 140 °C and 160 °C. Six CA measurements, each of a specific asphalt on a single aggregate at a given temperature, were performed. A goniometer (as in Figure 1) was used for the tests; in addition, it included an environmental chamber that ensured the required temperature was maintained. Figure 11 presents sample images obtained during the test.

**Figure 12:**
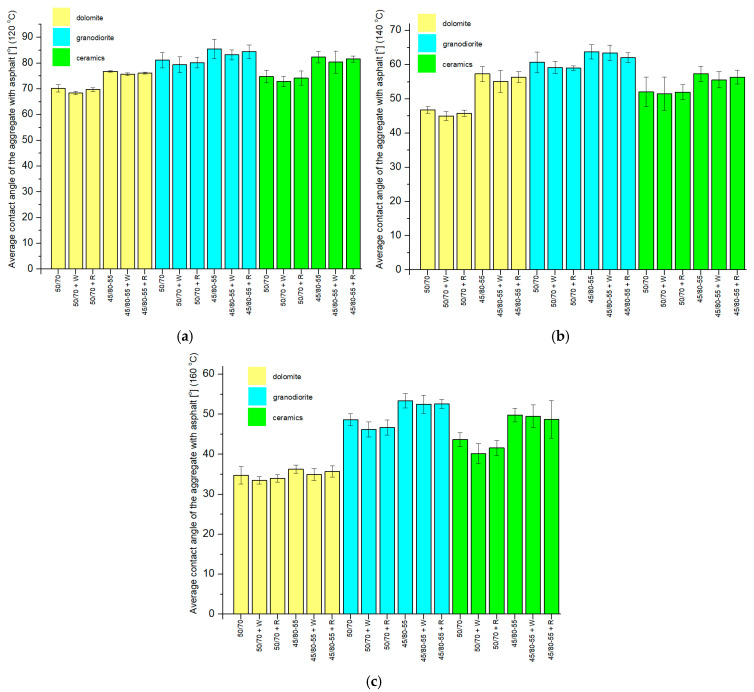
Mean CAs of aggregates with asphalt drops, along with standard deviations, at (**a**) 120 °C, (**b**) 140 °C, (**c**) 160 °C.

The graphs (Figure 12) show the obtained CA results with the asphalts. Calculations for the mean values and standard deviations of the samples were performed.

When analyzing the results of the CA measurements of aggregates with asphalts at a temperature of 120 °C, it can be observed that they are differentiated by the type of aggregate. The lowest mean CA was recorded for dolomite and 50/70 + W asphalt (68.4°) and was 20.0% lower than the highest mean CA obtained for granodiorite and 45/80-55 asphalt (85.5°). The mean CA of the ceramic aggregate with the asphalts used in the study adopted intermediate values. In addition, it can be noted that, within a particular aggregate, the mean CAs of ordinary asphalts (without and with additives) are smaller than the CAs obtained with the polymer-modified asphalts (without and with additives). This is probably due to the differences in the viscosity of the two types of asphalts at 120 °C.

When analyzing the results of the mean CAs of the aggregates and asphalt at 140 °C, there is a parallel with the results obtained from the CA measurements at 120 °C. The mean CA of the ordinary asphalts within a given aggregate was smaller than the mean CA for the polymer-modified asphalts. The mean CA obtained for the ceramic aggregate adopted intermediate values with respect to the CA of the respective dolomite and granodiorite samples. The lowest mean CA at 140 °C was obtained for dolomite and 50/70 + W asphalt (45.0°), and it was 29.5% smaller than the largest value, obtained for granodiorite and 45/80-55 asphalt (63.8 °C).

The results of the mean CA of the aggregates with asphalt in the third temperature group, i.e., 160 °C (Figure 12c), correspond with those obtained in the previous temperature groups—120 °C and 140 °C. A clear division in the dependence of the mean CA on the type of aggregate can be observed here. The smallest mean CA was recorded for dolomite and 50/70 + W asphalt (33.5 °C), which was 37.3% smaller than the largest mean CA obtained for granodiorite and 45/80-55 asphalt (53.4°). As in the other temperature groups, the ceramic aggregate obtained intermediate values for its mean CAs. The mean CAs within a single aggregate for the ordinary asphalts reached smaller values than the corresponding angles for the polymer-modified asphalts, although in the case of dolomite, the differences were insignificant.

When considering the dolomite aggregate, the smallest mean CA was obtained for asphalt 50/70 + W at 160 °C and was 54.4% smaller than the largest contact angle, obtained for asphalt 45/80-55 at 120 °C. In the case of the granodiorite aggregate, the smallest mean CA was read with asphalt 50/70 + W at 160 °C and was 46.0% smaller than the largest CA, obtained with asphalt 45/80-55 at 120 °C. In the case of the ceramic aggregate, the smallest mean CA was registered using asphalt 50/70 + W at 160 °C and was 51.2% less than the largest CA, obtained with asphalt 45/80-55 at 120 °C.

### 3.7. The Surface Free Energy (SFE) and Work of Adhesion (WA) in the “Aggregate–Water” and “Aggregate–Water/Glycerin” Systems

By determining the SFE and WA of the “aggregate–water” and “aggregate–water/glycerin” system, the characterization of aggregate top layers in terms of adhesion properties was carried out. It also enabled us to evaluate the behavior of the material when water was present.

The calculations of the total SFE values were carried out based on the CA measurements of the analyzed aggregates (Table 3). The calculations were carried out using two methods: the Neumann method [32] and the Owens–Wendt method [33]. The calculation assumed the most commonly used values of SFE and their respective components of measuring fluids in the literature.

SFE calculations were carried out using two methods, since the different methods for determining SFE based on the obtained CAs are based on different assumptions [49]. Therefore, the SFE values of the analyzed materials obtained using different methods and measuring fluids may not be equal [49,50]. Moreover, it was necessary to use two SFE calculation methods as a result of the differences observed in the findings presented by other authors in the scientific literature.

In addition, WA—work of adhesion—was determined based on the CAs with water and glycerin, respectively.

The highest value of SFE based on the mean CA with water, using the Neumann method, was calculated for granodiorite and was 55.66 mJ/m^2^. The lowest SFE value was obtained for the dolomite aggregate, it was 14.3% lower than the highest value and was 47.68 mJ/m^2^. The SFE of the waste ceramic aggregate took an intermediate value of 51.46 mJ/m^2^.

In the calculation of SFE, using the Owens–Wendt method, results similar to the SEP calculated by the Neumann method were obtained. The highest total SFE value was calculated for granodiorite and amounted to 58.95 mJ/m^2^, while the lowest value of total SFE was recorded for dolomite; it was 17.0% lower than the highest value and equaled 48.93 mJ/m^2^. As far as the components of the total SFE are concerned, the highest dispersive component of the SFE was obtained for ceramic aggregate—amounting to 38.7 mJ/m^2^—while the lowest value was calculated for dolomite and equaled 34.69 mJ/m^2^. In turn, the highest polar component of SFE, equal to 20.68 mJ/m^2^, was obtained for granodiorite, while the lowest value was obtained for dolomite and equaled 14.24 mJ/m^2^.

Comparing the two methods used to determine SFE, one can notice that the outcomes obtained using the Owens–Wendt method are greater than those obtained using the Neumann method for all the aggregates. Thus, the mean SFE value determined by the Owens–Wendt method for the dolomite aggregate was 2.6% higher than the mean SFE value obtained using the Neumann method; for granodiorite aggregate the difference was 5.6% and for recycled ceramic aggregate the difference was 6.1%.

In addition to calculating the SFE on this basis, the calculation of WA in the “aggregate–water” and “aggregate–glycerin” systems was also carried out. These calculations were based on the SFE values determined using the widely recognized Owens–Wendt method, since tests conducted with two liquids are more reliable than those using only one measuring liquid. The WA results were analogous to the SFE results. Thus, in the “aggregate–water” system, the largest WA value was calculated for granodiorite and was 122.72 mJ/m^2^, and the smallest was calculated for dolomite; it amounted to 108.89 mJ/m^2^ and was smaller than the largest by 11.3%. In the “aggregate–glycerin” system, on the other hand, the largest WA value was also calculated for granodiorite, it was 105.84 mJ/m^2^, and the smallest for dolomite; it was 93.60 mJ/m^2^ and was smaller than the largest by 11.6%. In both systems (“aggregate–water” and “aggregate–glycerin”), the WA for the ceramic aggregate reached intermediate values between those calculated for the other analyzed aggregates.

Since higher SFE values were obtained using the Owens–Wendt method than the Neumann method, indicating better adhesion of water and glycerin and higher WA, the less favorable variant was adopted for further analysis.

### 3.8. The Determination of the Adhesion of the Tested Asphalts to the Aggregates Using the Pull-Off Method

Pull-off tests were performed to evaluate the strength of adhesion between the gravel and binder. Following the pull-off test, the peel-off surface was examined, which was necessary to determine the type of damage (adhesion damage, cohesion damage) and important for evaluating the results obtained in this experiment. To determine the maximum stress in the pull-off test on the basis of the radius of the steel ring (r = 25.00 mm), its surface area was taken as 1963.50 mm^2^.

**Figure 13:**
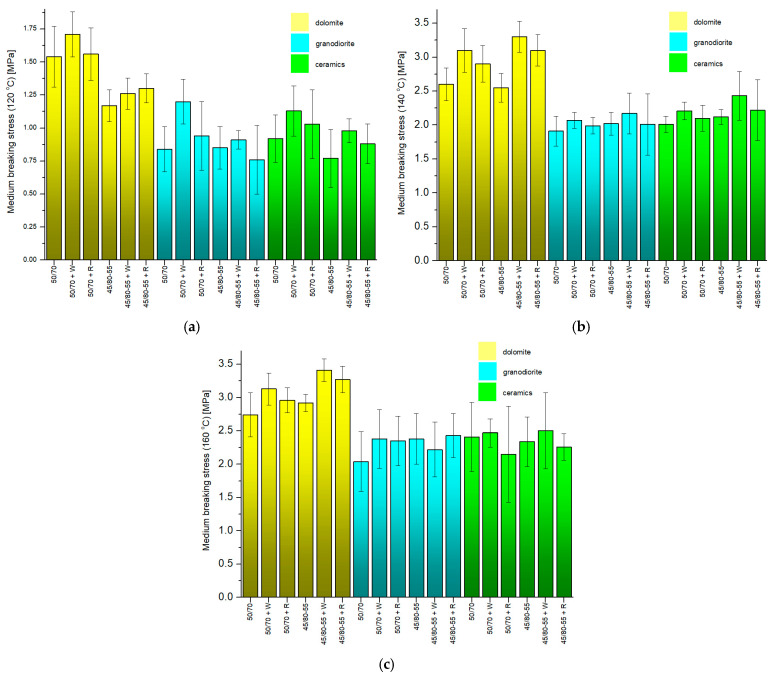
The mean pull-off stresses obtained in the pull-off tests with different temperatures of the asphalts when applied to the aggregates: (**a**) 120 °C, (**b**) 140 °C, (**c**) 160 °C.

Figure 13 shows the pull-off test results.

Within the samples with a dolomite aggregate, the lowest average stress (1.17 MPa) was obtained using 45/80-55 modified asphalt without adhesives at 120 °C and was 65.7% lower than the highest value (3.41 MPa) obtained for samples with the 45/80-55 + W modified asphalt at 160 °C. Within the samples with the granodiorite aggregate, the smallest average stress (0.76 MPa) was obtained using 45/80-55 + R modified asphalt at 120 °C and was 68.7% less than the largest value (2.43 MPa) obtained for samples with the 45/80-55 + R modified asphalt at 160 °C. As for the waste ceramic aggregate, the smallest average stress (0.77 MPa) was obtained using asphalt modified 45/80-55 without adhesives at 120 °C, and it was 69.2% lower than the highest value (2.50 MPa) obtained when using samples with asphalt modified with 45/80-55 + W at 160 °C.

## 4. Discussion

### 4.1. The Analysis of the Effects of Aggregate, Asphalt Characteristics and Temperature on the Adhesion of Asphalt and Aggregate

#### 4.1.1. Comparative Analysis

The comparative analysis in the context of checking how the factors of aggregate, asphalt and temperature affect the asphalt and aggregate adhesion took the form of a multivariate analysis of variance (MANOVA). This took into account the fact that adhesion consists of two factors: the CAs of the aggregates with the asphalts and the adhesion as determined by the pull-off tests. The asphalts were characterized as a function of viscosity. The characteristics of the aggregates were expressed by the 3D roughness parameter in terms of the arithmetic mean surface height Sa. Unfortunately, both the assumption of normality and homogeneity of the covariance matrix were not satisfied.

Although the parametric MANOVA test could be used, there are permutation equivalents of the MANOVA test that do not have such stringent assumptions. The only assumption of the test is that the distributions in each subgroup should be of a similar spread, which was met.

The permutation technique involves permuting the predictors (independent variables) and observing the effect of this action on the values of the dependent variable (or variables, in this case). It can be expected that if there is a relationship between the independent and dependent variables, the values in the sample will confirm this relationship. On the other hand, if we swap the values of the independent variable while leaving the dependent variable unchanged, then this relationship will no longer be reflected in the permuted sample. Ultimately, testing with this method involves checking how often the value of the test statistic for the permuted samples will be greater than the value of the test statistic for the original data (sample).

Investigating the highest-order interaction, that is, the aggregate–asphalt–temperature interaction, is not possible, because there is only one value of contact angle and pull-off adhesion (the average of six measurements) in the intersection of the values of these variables. Therefore, the second-order interactions were examined first.

The test results obtained did not give grounds to reject the hypothesis of an interaction of two factors, which made us reject a second-order interaction (only one, since it could be that after removing one insignificant interaction, another one would become significant).

Since subsequent tests (after each removal of a second-order interaction) showed that the second-order interactions were not significant, only additive effects were left in the model (Table 4).

All the effects are statistically significant. Considering the value of the partial R^2^, it can be concluded that of the three effects analyzed, temperature is the most significant. However, only post hoc analysis can show this. Since interaction effects are not statistically significant, only additive effects (each separately) were considered in the post hoc analysis.

The characteristics of adhesion, as indicated by the pull-off adhesion and the cohesion of aggregate with asphalt, exhibit statistically significant variations that are dependent upon the employed aggregate type. The graphs of the individual dependent variables show these differences.

The comparison of asphalts showed only one pair differing in the context of adhesion, namely asphalt type 50/70 differing significantly from asphalt type 45/80-55 + W. Also on the borderline of significance are the pairs 50/70 and 45/80-55 + R as well as 50/70 + R and 45/80-55 + W. In contrast, a comparison of adhesion by temperature shows highly significant differences between all analyzed pairs.

**Figure 14:**
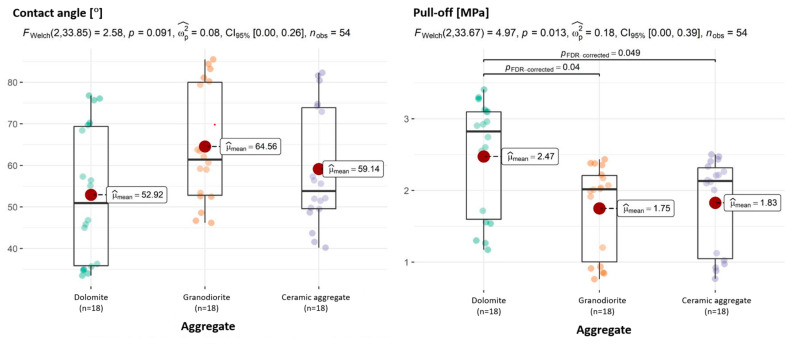
Relationships between aggregate and CA of aggregate–asphalt and aggregate and adhesion (pull-off), with determination of statistically significant differences.

The analysis of individual adhesion dimensions by aggregate type provides interesting results. While the post hoc test detected differences in adhesion for every pair of aggregates compared, single-dimension tests yield less obvious results. As was mentioned earlier, multi-dimensional tests can detect differences that one-dimensional tests do not. Thus, only in terms of adhesion (pull-off) are there differences between dolomite and granodiorite and dolomite and ceramic aggregate (Figure 14). In terms of the CAs of aggregate with asphalt, no statistically significant differences were observed (Figure 14).

**Figure 15:**
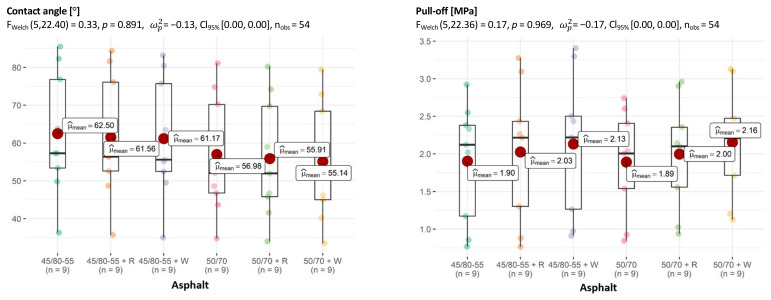
Relationship between asphalt and CA of aggregate–asphalt and adhesion (pull-off), with determination of statistically significant differences.

The situation in the asphalt breakdown also seems interesting. While in the multivariate test the effect of asphalt was significant and there was at least one pair for which the differences in adhesion were significant, the one-dimensional tests do not confirm this (Figure 15).

**Figure 16:**
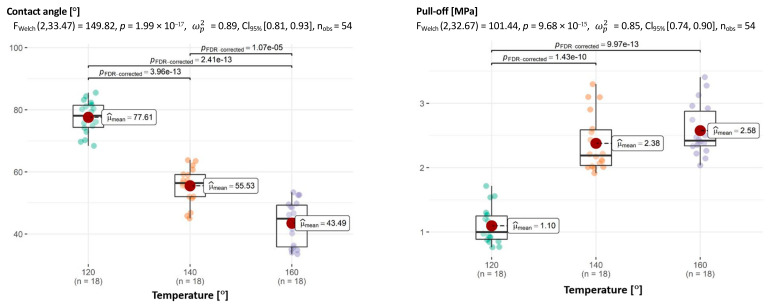
Relationship between temperature and CA of aggregate–asphalt and temperature and adhesion (pull-off) with determination of statistically significant differences.

As far as the effects of temperature on adhesion is concerned, the picture drawn from the analysis of individual dimensions is similar to that drawn from multivariate tests. Except for one pair (140 °C and 160 °C for pull-off), all other comparisons are significant (Figure 16).

In summary, the outcomes of the statistical tests conducted indicate that the findings from the permutation equivalent of the MANOVA test have been validated.

#### 4.1.2. Cluster Analysis

In order to determine groups of observations that were homogeneous by adhesion parameters (in this case, adhesion consists of two factors: the contact angle of the aggregates with the asphalt and adhesion as determined by the pull-off test), cluster analysis was conducted. The first stage of this analysis involved the application of a hierarchical cluster analysis, and on this basis the optimal number of clusters was extracted. A division into six clusters was considered in the conducted analyses. The second stage involved using the k-means method to divide the observations into homogeneous groups. The results of the clustering were presented on a dendrogram and a projection plot on the space defined by the principal components using the PCA method.

For cluster analysis, the following code algorithm was adopted for the designation of individual observations:

- The first letter of the code corresponds to the type of aggregate (D—dolomite; G—granodiorite; K—ceramic aggregate);
- Then, there is a code for the asphalt used (the first digit of the asphalt designation and the possible addition of “W” or “R” if there is a Wetfix BE or Rediset asphalt adhesion agent, such as 50/70 + W—“5W” or 45/80-55 + R—“4R”);
- The final three characters of the code are the temperature value in degrees Celsius at which the observation took place.

**Figure 17:**
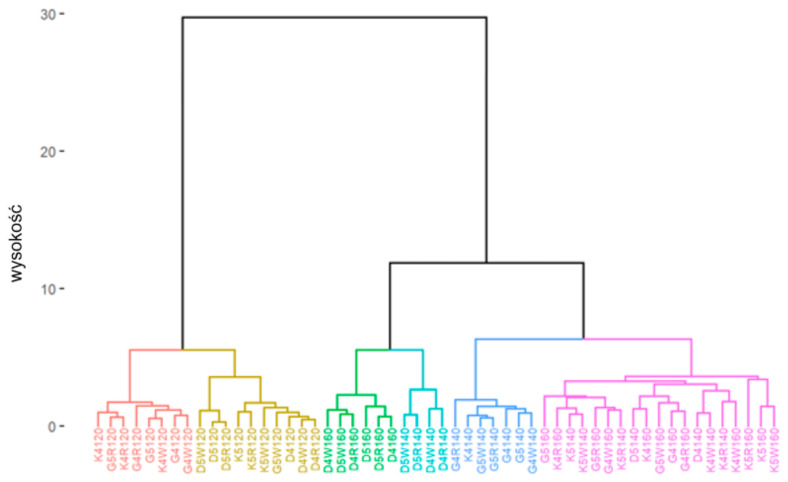
Dendrogram of individual observations when divided into six clusters.

**Figure 18:**
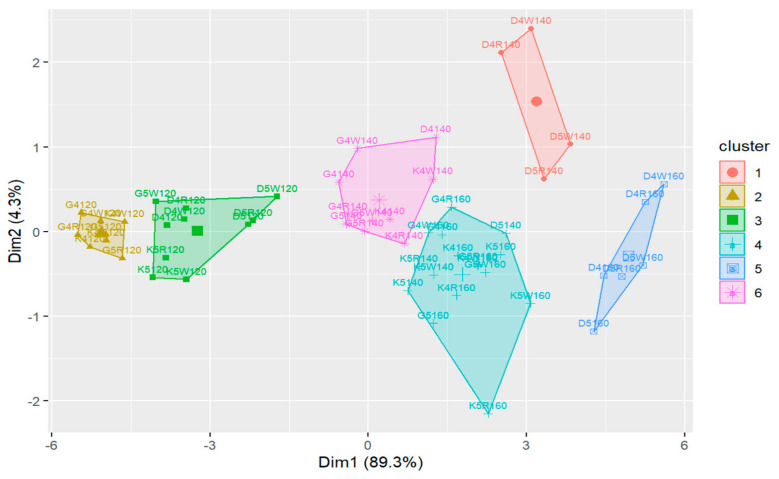
The projection of the observations on the space defined by the principal components in the case of division into six clusters.

The cluster analysis outcomes obtained for the division into six clusters are shown below (Figure 17 and Figure 18).

Considering the obtained results of the cluster analysis with the division into six clusters, it can be seen that temperature constitutes the primary factor that determines the clustering of the observation data, which, in addition, was confirmed by the comparative analysis conducted earlier in Section 4.1.1. The projection retained 93.6% of the information from the individual observations.

### 4.2. Adhesion

#### 4.2.1. Affinity Between Aggregates and Asphalts

The results obtained in the study of the affinity between asphalts and aggregates directly correspond with the findings related to the chemical composition of aggregates and their surface roughness. This is most evident in the affinity between aggregates and asphalts without adhesives (50/70, 45/80-55). This affinity is highest for dolomite—an alkaline rock with the most strongly developed grain surface texture—and amounts to 65–70%, governed by asphalt type (Figure 8). The weakest, most unsatisfactory affinity was obtained for the aggregate with a high silica content and characterized by the least developed surface texture—ceramic aggregate. A slightly higher affinity for asphalt was shown by granodiorite aggregate. Zięba M. and Lewandowska W. [51] obtained similar results for the affinity of asphalt to aggregates with different silica content, determined on the basis of PN-EN 12697-11 method A. Sybilski and Błażejowski indicated that dolomite was characterized by a greater affinity for asphalt following modification with the PMB 45/80-55 polymer, achieving a rate of 85%, compared to an affinity of 80% for granite [5,52]. In the present study, these values are lower for dolomite by a mean of 17.6%, and for granodiorite by 31.2%. In addition, Zięba M. and Witczak W. suggested, based on their experiments, that the type of asphalt used for testing in line with PN-EN 12697-11, method A, influences the percentage of aggregate coverage with asphalt. This conclusion corroborates the findings obtained in affinity studies between aggregates and the asphalts analyzed [53]. A significantly higher percentage of aggregate surrounding with asphalt in the affinity determination test was recorded for samples with PMB 45/80-55-modified asphalt. According to the results of this study, when dolomite, granodiorite or sanitary ceramics are employed in mineral–asphalt mixtures, adhesion agents should be utilized to enhance the asphalt-to-aggregate adhesion.

The addition of adhesion agents to asphalts in the amount of 0.3% by weight of asphalt clearly increased the value of the mean affinity between the asphalts and aggregates analyzed in the study. The analysis revealed that, in several instances, the aggregate and asphalt adhesion reached a minimum of 80% following six hours of the “rotating bottle” test. The most pronounced increase was observed for acidic aggregates, where the adhesive agents acted as activators of asphalt adhesion to the surface of the aggregate.

The majority of aggregate surfaces are characterized by a lack of electrical inertness [44]. As a result of the presence of oxygen atoms on the aggregate surface that are not entirely electrically inert, silica, which is the primary constituent of magmatic rocks, possesses a slight negative charge [54]. The adhesion results from the interaction of relatively weak, diffuse electrical charges [54]. Acidic aggregates demonstrate greater hydrophilic characteristics compared to basic aggregates [55,56]. Asphalt, constituting an electroneutral colloid, exhibits superior adhesion to alkaline, electroneutral aggregates, compared to acidic ones [27].

#### 4.2.2. CA of Aggregates with Water and Glycerin

In order to evaluate the results obtained in the CA study of aggregates with water and glycerin, reference was made to the literature. A comparable study was performed by Hou Y. et al. [6,57]. Among other things, they used a drop of water to measure the CAs of selected aggregates. According to the results obtained, it may be inferred that water wetted the aggregate with a high silica content (granite) best, while the aggregate with a low SiO_2_ content (limestone) was the worst. An analogous relationship was observed in the results of the experiment presented in the article; water-wetted granodiorite was the best (smallest CA) and dolomite was the worst (largest CA). In line with the theory of chemical adhesion, it is possible to classify aggregates as hydrophilic (attracting water) and hydrophobic (repelling water). In general, hydrophilic aggregates, e.g., siliceous aggregates, which are most often acidic (such as granodiorite), form weak adhesion bonds with asphalt. It is easier for asphalt to be stripped from their surface than is the case with more hydrophobic aggregates. This is related to the damage resistance of MAMs.

Hou Y. et al. [6] presented the results of wetting asphalt with a drop of water. The contact angle (CA) value observed between asphalt and water was approximately 90°, suggesting that these two materials possess distinct characteristics and exhibit limited compatibility. Furthermore, it indicates that water has a higher polarity compared to asphalt. On the basis of this observation, Hou Y. et al. proposed that due to the contrasting polarities of water and asphalt, materials that demonstrate good wettability with water tend to exhibit poor wettability with asphalt. In order to confirm the latter suggestion, in Section 3.6, CA determination for the aggregates with asphalt was carried out. The results confirmed that the asphalt better wetted those aggregates that were more poorly wetted by water.

#### 4.2.3. CA of Aggregates with Asphalt

On the basis of the study, it can be concluded that the smallest mean CA was recorded for dolomite and 50/70 + W asphalt at 160 °C (33.5°), and it was 60.8% lower than the highest observed CA for granodiorite and 45/80-55 asphalt at 120 °C (85.5°). It should be noted that the best wettability of an aggregate with asphalt was obtained for the alkali aggregate (dolomite) and the asphalt with the lowest mean softening temperature (Table 3). Similarly, the sample consisting of the aggregate characterized by the highest content of silica (granodiorite) and asphalt with the highest mean softening temperature showed the weakest wettability. It is noteworthy that as the test temperature increased, the mean CAs of the aggregates with asphalt reached lower and lower values, and this was true for all the analyzed aggregates and asphalts. This aspect should be associated with a critical parameter that significantly impacts the aggregate and aggregate adhesion, namely the asphalt viscosity. Viscosity, which quantifies the cohesion among hydrocarbon molecules in the bituminous substance, is mainly influenced by the existence of particular functional groups, as well as temperature [47]. With increasing temperature, the binder viscosity reduces, thereby promoting the easier wetting of the aggregate grain surface.

Furthermore, the wetting characteristics exhibited by asphalt can be elucidated through the surface energy theory [58]. Upon contact between aggregate and asphalt, an adhesive tension is established, which typically exceeds that between water and the aggregate compared to a binder and the aggregate. Consequently, when water is present, asphalt tends to be displaced from the aggregate’s surfaces, hindering proper wetting. This process facilitates the leaching of the asphalt layer that coats the aggregate. [59,60].

Very similar conclusions on the basis of conducted studies were presented by Yao H. and others [61]. The researchers conducted MD simulations to analyze the CAs between asphalt and aggregates. They employed droplet shape analysis to evaluate the images and ascertain the CAs. Additionally, the CAs between the binder droplets and the aggregates were measured using a goniometer. While analyzing the conducted tests, they observed that in the interfacial asphalt–aggregate system large CAs occurred at lower temperatures, whereas small CAs were observed at elevated temperatures. As the temperature rose, the interface between the mineral material and the binder shifted from partial non-wetting to a state of complete wetting.

#### 4.2.4. Surface Free Energy and Work of Adhesion

During the analyses, it was noted that A. Naveed [62] obtained SFE values of granite (60.0 mJ/m^2^) very close to the SFE obtained for granodiorite (55.6 mJ/m^2^ and 58.95 mJ/m^2^) in the studies conducted. Similarly, Fic S. [10] showed that the SFE results obtained for dolomite (45.6 mJ/m^2^) and medium-grained granite (59.4 mJ/m^2^) corroborate those presented in this study for dolomite and granodiorite. In addition, Hou Y. et al. k [6] determined the SFE for aggregates with low (limestone) and high (granite) silica contents. The alkaline aggregate (limestone) obtained a lower SFE than the acidic aggregate (granite). A similar relationship was found in the SFE studies presented in Section 3.7; dolomite with a low proportion of SiO_2_ in its composition had a lower surface energy compared to the ceramic aggregate and granodiorite, i.e., aggregates with a high SiO_2_ content. It is evident that the SFE values of aggregates largely depend on the place of origin of these aggregates and the research methodology adopted.

The high SFE value of the water–aggregate system may suggest a greater susceptibility of the aggregate to the stripping of the binder from its surface in the mineral–asphalt mixture. A water–aggregate system with a low SFE value indicates that in an asphalt–aggregate system using the same aggregate, the potential for the binder to be washed off the surface of the aggregate will be lower, and thus it will be characterized by higher resistance to water- or moisture-induced damage [9,60]. In the zone of contact between the aggregate surface and water, the molecules of water strive to realize the energy demand through the aggregate surface. Due to their dipolar nature, molecules of water are more capable of meeting the energy requirements of the aggregate surface, which predominantly consists of non-polar molecules found in asphalt. This interaction results in a reduction in the adhesion forces between materials and asphalt in MAMs. The extent of this phenomenon is significantly influenced by the surface composition of the aggregate grains [63,64].

#### 4.2.5. Pull-Off

While examining the findings from the pull-off test, it can be inferred that at all the tested asphalt application temperatures on the aggregates, the highest mean adhesion values were obtained for dolomite and the lowest for granodiorite, with the mean adhesion values of asphalt to the ceramic aggregate being slightly higher than those obtained for granodiorite. The granodiorite aggregate achieved the lowest peel adhesion force values of all the aggregates in most cases. This may be due to its lower roughness and high-percentage SiO_2_ content, which, as a relatively acidic material, reduced the asphalt-to-aggregate adhesion [65]. A similar suggestion was made by Ji X. et al. on the basis of the results of the adhesion strength of asphalt to selected aggregates (basalt, limestone, diabase, granite) determined by the pull-off test in their study [66].

In addition, it is possible to notice an increase in the mean stress in the pull-off test as the temperature of application of the asphalts to the aggregates increases. A clear difference can be seen, especially between 120 °C and 140 °C. Also noteworthy is the observed trend whereby most of the samples tested with PMB 45/80-55-modified asphalt exhibit higher adhesion to the aggregate than the samples with 50/70 ordinary asphalt. In addition, considering the obtained adhesion results related to a single aggregate, it may be observed that the mean stress values in the pull-off test are higher for samples where asphalts with adhesion agents were used. The substances used modify the chemical–physical form of contact between the binder and aggregate. The occurrence of these substances causes a decrease in the surface tension of the asphalt, thereby enhancing the wettability of the aggregate with the asphalt. Most of the samples in which the Wetfix BE adhesion agent was added to the asphalt obtained the highest mean adhesion values. Thus, for example, at 140 °C, all the analyzed aggregates recorded the highest adhesion values with asphalt 45/80-55 + W, with the following values: dolomite—2.30 MPa; granodiorite—2.17 MPa; ceramic aggregate—2.43 MPa.

## 5. Conclusions

Following the laboratory tests, the obtained findings and the statistical evaluations of the tested aggregates and asphalts, the subsequent final conclusions have been formulated:

- The tested aggregates showed notable differences in micromorphology, roughness and surface free energy (SFE) due to their distinct chemical compositions and diagenetic conditions;
- Adhesion between aggregate and asphalt depends on microscopic chemical and physical interactions, influenced by aggregate surface chemistry, micromorphology and asphalt properties;
- Higher surface roughness enhances adhesion, making it a crucial factor in aggregate selection;
- Temperature strongly affects adhesion, with aggregate roughness playing a secondary role and asphalt type having minimal impact, though temperature significantly influences asphalt viscosity;
- Aggregates characterized by more favorable wettability with water (lower CA with water) showed poor wettability with asphalt (higher CA with asphalt). Hydrophilic aggregates (e.g., siliceous types) exhibit weaker adhesion with asphalt due to their polarity, making asphalt more prone to stripping;
- A higher SFE in the water–aggregate system increases binder removal risk, while a lower SFE suggests better moisture resistance;
- The use of asphalts with the addition of an adhesive agent has a positive effect on the adhesion between the asphalt and the aggregate, described by the contact angle of the aggregate with the asphalt and adhesion (“pull-off”).

The directions for further research are as follows:

- Determining the viability of incorporating recycled ceramic aggregate into MAMs intended for wearing surfaces;
- Determining the effect of the mineral mixture compositions and asphalt varieties utilized on the properties of asphalt concrete intended for wearing surfaces, in terms of the service life of the pavement.

## Figures and Tables

**Table 1:** Selected parameters of studied asphalts.

Type of Asphalt	Penetration of Asphalts [0.1 mm] According to PN-EN 1426			Softening Temperature of Asphalts [°C] According to PN-EN 1427		
	Mean	SD	CV [%]	Mean	SD	CV [%]
50/70	61.144	0.577	0.9	51.067	0.242	0.5
50/70 + R	63.500	0.702	1.1	50.767	0.234	0.5
50/70 + W	64.644	0.958	1.5	50.667	0.327	0.6
45/80-55	51.767	0.939	1.8	72.333	0.242	0.3
45/80-55 + R	51.244	0.906	1.8	70.233	0.197	0.3
45/80-55 + W	52.856	0.265	0.5	69.967	0.234	0.3

W—adhesion agent described in point 2.2.1, Wetfix BE; R—adhesion agent described in point 2.2.2, Rediset LQ-1102CE.

**Table 2:** Selected parameters of studied aggregates.

Property			Dolomite	Granodiorite	Ceramic Aggregate
specific density [g/cm^3^]		mean	2.60	2.67	2.64
bulk density [g/cm^3^]		mean	2.42	2.61	2.40
absorptivity [%]		mean	1.1	0.4	1.4
shape index SI [%] PN-EN 933-4		mean	4	0	20
		SI category	SI_10_	SI_10_	SI_20_
flatness index FI [%] PN-EN 933-3		mean	5	1	16
		FI category	FI_10_	FI_10_	FI_20_
LA Abrasion [%] PN-EN 1097-2	Los Angeles value	mean	23	16	22
	LA category		LA_25_	LA_20_	LA_25_
frost resistance F [%] PN-EN 1367-1	freezing—thawing; percentage weight loss	mean	0.6	0.1	0.2
	F category		F_1_	F_1_	F_1_

**Table 3:** SFE calculated using Owens–Wendt method for “aggregate–water/glycerin” system and work of adhesion.

Aggregate	Mean CA Using Water [°]	Mean CA Using Glycerin [°]	SEP—Neumann Method [mJ/m^2^]	SEP—Owens–Wendt Method [mJ/m^2^]			Work of Adhesion W_A_ [mJ/m^2^]	
			SEP	SEP Dispersive Component	SEP Polar Component	Total SEP	Work of Adhesion W_A_ (Water)	Work of Adhesion W_A_ (Glycerin)
Dolomite	60.28	60.47	47.68	34.69	14.24	48.93	108.89	93.60
Granodiorite	46.71	46.52	55.66	38.27	20.68	58.95	122.72	105.84
Ceramic aggregate	53.96	54.13	51.46	38.47	16.33	54.80	115.63	99.44

**Table 4:** The results of the conducted tests for the significance of the additive effects.

Effect	R^2^	*p*-Value
**Aggregate**	0.17022	0.0001 (1 × 10^−4^)
**Asphalt**	0.14195	0.0001 (1 × 10^−4^)
**Temperature**	0.41663	0.0001 (1 × 10^−4^)

## Data Availability

Data are contained within the article.
